# CYSTOGASTROSTOMY WITH ARGON PLASMA COAGULATION PROBE AND WITHOUT ENDOSCOPIC ULTRASONOGRAPHY

**DOI:** 10.1590/0102-672020180001e1444

**Published:** 2019-08-26

**Authors:** Omer Faruk OZKAN, Erdem AKBAL, Sukru TAS, Fahri GUNES

**Affiliations:** 1 Department of General Surgery;; 2 Department of Gastroenterology;; 3 Department of Internal Medicine, Çanakkale Onsekiz Mart University, Çanakkale, Turkey.

**Keywords:** Argon Plasma Coagulation, Ultrasonography, Gastrostomy, Coagulação com Plasma de Argônio, Ultrassonografia, Gastrostomia

## INTRODUCTION

Acute pancreatitis is an inflammatory condition of the pancreas which can lead to morbidity. Formation of pancreatic pseudocyst is one of the well-known complication. While small pseudocyts are asymptomatic, large ones can become symptomatic and cause several complications including infection, rupture, bleeding, biliary complications and portal hypertension^1^ ^,^ ^2^.

 Various interventions are available for the management of symptomatic pancreatic pseudocysts. Endoscopic ultrasound (EUS) guided cystogastrostomy is a choice for treatment of large pseudocyts, witch bulge into gastric lumen^2^ ^,^ ^3^. In this paper we present a case of large sized who was managed with argon plasma coagulation probe and without endoscopic ultrasonography.

## CASE REPORT

**FIGURE1:**
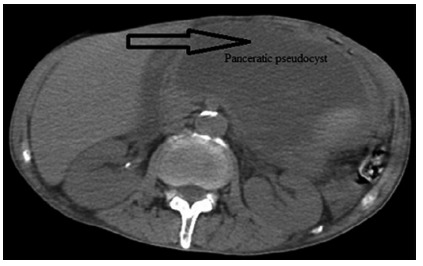
Pancreatic pseudocyst with 150x100 mm pushing gastric wall

Fifty years old male was in reanimation clinic with the diagnosis of complicated and severe acute pancreatitis due to gallstones for three months. His physical examination revealed a large sized mass extending from epigastric to left upper quadrant of abdomen. The contrast enhanced CT showed a cystic lesion with 150x100 mm dimensions in the tail and body of pancreas pushing the stomach (Figure1). The diagnostic upper gastrointestinal endoscopy revealed a bulge localized on large curvature related to pancreatic pseudocyst. An endoscopic cystogastrostomy was planned. After detection of the area for cytogastrostomy in gastric lumen with standard video-endoscope (Pentax EG 290 LK), it was marked by argon plasma coagulation probe (30 watt); the gastric wall was opened step-by-step with the probe (60 watt) until pancreatic fluid drainage into stomach was seen. After aspiration of pancreatic fluid (approximately 1500 ml), the gastric opening area enlarged by using an ERCP sphincterotomy. Then a guide wire was inserted into the cyst with the C arm fluoroscopy. Finally, 8.5 F pigtail plastic stent was placed into the cyst through the gastric lumen. The procedure was completed without any complication. 

**FIGURE 2:**
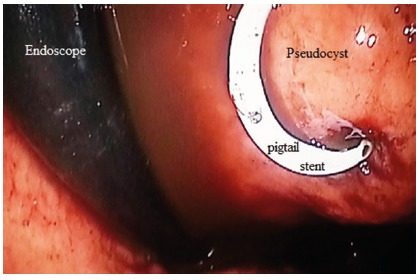
Plastic pigtail stent installed with argon plasma coagulation probe cut

## DISCUSSION

EUS guided cystogastrostomy is a safe method for management of pancreatic pseudocyts^1^ ^,^ ^3^. EUS assisted cytogastrostomy has a significant advantage by providing relation between cyst wall and gastric wall, cyst fluid imaging features, and gastric wall vessels^2^. If pseudocysts have a bulge through the gastric lumen, cytogastrostomy can be performed without EUS. To avoid the complications such as bleeding, cystogastrostomy was performed by an argon plasma coagulation probe^2^ ^,^ ^3^. In the literature, EUS with cytogastrostomy procedures usually performed with needle knife and YAG laser ^4^.

Our case has demonstrated that argon plasma coagulation without endoscopic ultrasonography cytogastrostomy can be an option in handling large volume pancreatic pseudocyst during endoscopic cystogastrostomy. 
